# Describing the status quo of person-centred dementia care in different types of care units in German nursing homes: A convergent mixed methods study

**DOI:** 10.1016/j.ijnsa.2024.100233

**Published:** 2024-08-10

**Authors:** Anna Louisa Hoffmann-Hoffrichter, Mike Rommerskirch-Manietta, Johannes Michael Bergmann, Martina Roes, Bernhard Holle, Rebecca Palm

**Affiliations:** a German Center for Neurodegenerative Diseases (DZNE), Stockumer Str. 12, 58453 Witten, Germany; b Witten/ Herdecke University (UW/H), Faculty of Health, Department of Nursing Science, Alfred-Herrhausen-Straße 50, 58448 Witten, Germany

**Keywords:** Convergent mixed methods design, Dementia, Mission statements, Nursing homes, Policy, Person-centred care

## Abstract

**Background:**

The policies and mission statements of nursing homes support the implementation of person-centred dementia care. The Dementia Policy Questionnaire assesses the content of person-centred dementia care in policies. To date, it is unknown whether these policies exist exclusively in dementia care units and whether the policies are consistent with the mission statements of nursing homes.

**Objective:**

We aimed to (1) investigate nursing home care unit types regarding the existence of policies measured by the Dementia Policy Questionnaire, (2) explore whether these policies are addressed in the mission statements of the nursing homes, and (3) integrate both results.

**Design:**

This is a convergent mixed methods study performed with a quantitative and qualitative dataset that was collected in the BeStaDem survey (2020).

**Setting:**

The BeStaDem survey included licensed nursing homes in Germany.

**Participants:**

A total of 134 nursing home administrators provided informed consent to participate in the BeStaDem survey.

**Methods:**

For quantitative data, we performed Fisher's exact test to identify differences in the Dementia Policy Questionnaire item distribution of several types of care units (aim 1). To support the results of Fisher's exact test, we additionally applied logistic regression analysis. For qualitative data, we analyzed the mission statements deductively with the qualitative content analysis method (aim 2). For integration, we used a convergent triangulation approach (aim 3).

**Results:**

The quantitative data collected from 134 German nursing homes show significant associations among person-centred dementia care policies, such as behavior assessment, and nursing homes with dementia care units. Regarding the qualitative data, of the 60 mission statements in total, eight mission statements of nursing homes with dementia care units exclusively address aspects such as dementia-specific interventions. The convergent triangulation approach shows that the answers given by the nursing homes in the quantitative survey are not always consistent with what they address in their mission statements.

**Conclusions:**

Nursing homes with dementia care units provide more person-centred dementia care policies than other care unit types do but mostly do not address these aspects in their mission statements. The implementation of person-centredness benefits from the existence of policies and mission statements if nursing homes clearly address what is meant by person-centred dementia care in their nursing home.

**What is already known**

• Nursing homes provide mission statements and internal policies as a form of management support for staff.
• Regarding person-centred dementia care, on the one hand, policies must meet quality requirements; on the other hand, they must be flexible with respect to the preferences of residents.

Alt-text: Unlabelled box

**What this paper adds**

• Policies regarding person-centred dementia care differ across different types of care units in nursing homes.
• Dementia Care Units and Dementia Special Care Units provide more policies regarding person-centred dementia care.
• Nursing homes should provide internal policies that are consistent with the objectives addressed in their mission statements.

Alt-text: Unlabelled box

## 1 Background

Since people living with dementia form the largest group of people living in nursing homes (Hoffmann et al., 2014), person-centred dementia care is gaining importance worldwide (World Health Organization, 2017). The goal of person-centred dementia care is to maintain personhood by supporting people's autonomy, fostering and maintaining relationships, and satisfying psychosocial needs (Hennelly et al., 2019; Kitwood, 2019). To implement person-centred care in long-term care, formal structures are integrated into the respective organization (Kühl, 2020; Nettelstroth, 2004).

Organizational structures affect decisions within an organization and can be understood as the working conditions for employees (Kühl, 2020). Formal organizational structures include organizational regulations that relate to the tasks and activities of staff to achieve organizational intentions (Mariani et al., 2017) and are expressed in procedural guidelines (Nettelstroth, 2004) as well as requirements for work processes and standards (Bartholomeyczik, 2002). In Germany, the terms regulations, standards, and policies are used synonymously within an organization. In this article, we define policies as an umbrella term for organizational regulations. In German nursing homes, quality managers or nursing home administrators develop policies at the management level.

Staff can experience policies as a form of management support that provides common ground and reinforces their ability to perform person-centred care interventions (Mariani et al., 2017). In long-term care policies, it is necessary to find a balance among protection against risks for people living with dementia, flexibility and consideration of their needs and wishes (Carr and Biggs, 2020).

Nursing home policies include content about care activities, and they guide how the organization achieves its objectives. Policies should be consistent with the purpose of an organization (Hoyle, 2017). Mission statements incorporate an organization's purpose (Hoyle, 2017), philosophy and values, mission and vision (Braun et al., 2012). Quasdorf and Bartholomeyczik (2017) identified the provision of a person-centred vision anchored in a nursing home mission statement as guidance for person-centred dementia care. Therefore, we argue that a nursing home mission statement should include a person-centred vision.

To assess whether and which policies about person-centred dementia care exist in German nursing homes, we developed the Dementia Policy Questionnaire. The Dementia Policy Questionnaire includes 14 items that have been forward translated and culturally adapted from items on the Assessment of Policies for Person-Centered Management of Behavioral and Psychological Symptoms of Dementia (BPSD) (Resnick et al., 2020) as well as five items developed based on reviewed literature (Hoffmann et al., 2022). The items assess the existence of policies about the assessment of a person's preferences, shared decision making and dementia-specific interventions (Table 1). The development of the Dementia Policy Questionnaire has been described elsewhere (Hoffmann et al., 2022). After testing the preliminary Dementia Policy Questionnaire, the results indicate further development of the instrument regarding (1) items that are specific for dementia special care in German nursing homes, (2) further aspects of person-centred dementia care, and (3) construct validity. To further develop the preliminary Dementia Policy Questionnaire, it is useful to investigate whether preliminary Dementia Policy Questionnaire items exist exclusively in care units specializing in dementia care in nursing homes using a mixed-methods approach.

**Table 1:** Items and abbreviated names according to Hoffmann et al. (2022).

No.	Item	Categories	Abbreviated name
***Subdimension: Internal policies regarding recording residents’ preferences***			
1	There are established visitor regulations that regulate visiting times and the number of visitors.	No	Visitor 0
		Yes	Visitor 1
2	The preferences of residents are recorded systematically and in a structured way.	No	PreferenceA 0
		Yes	PreferenceA 1
3	The hospitalization transfer form includes information regarding residents’ preferences.	No	Sheet 0
		Yes	Sheet 1
4	There is a (written) procedure that includes residents in the staff selection process when employed.	No	Selection 0
		Yes	Selection 1
5	There is a (written) procedure that specifies that residents or their legal representatives (relatives) are to participate in case conferences.	No	CConference 0
		Yes	CConference 1
6	There is written policy regarding the manner in which nursing assistants are included in case conferences.	No	Involvement 0
		Yes	Involvement 1
7	There is a written policy that provides residents with all-day use of common areas.	No	Area 0
		Yes	Area 1
8	Residents' preferences, which should be taken into account when performing prophylaxis, are recorded systematically and in a structured manner.	No	PreferenceB 0
		Yes	PreferenceB 1
9	There is a policy regarding the manner in which external employees without regular access to nursing documentation (such as cafeteria service staff or external service providers) receive information concerning residents’ preferences.	No	PreferenceC 0
		Yes	PreferenceC 1
***Subdimension: Internal policies regarding participatory decision making***			
10	There is a policy that stipulates that upon moving into the nursing home, a conversation regarding care planning is held with the resident and family members.	No	CarePlan 0
		Yes	CarePlan 1
11	There is a policy that outlines the manner in which residents and family members are to be involved in procedures used as an alternative to restraint.	No	AltRestrict 0
		Yes	AltRestrict 1
12	There is a policy regarding ways of dealing with refusals of nursing interventions and prophylaxis.	No	Rejection 0
		Yes	Rejection 1
***Subdimension: Internal policies regarding dementia-specific interventions***			
13	The number of residents taking psychotropic drugs or neuroleptics is regularly evaluated as part of internal quality management.	No	Drugs 0
		Yes	Drugs 1
14	Dementia-specific instruments are used to assess pain.	No	Pain 0
		Yes	Pain 1
15	Dementia-specific behavioral assessment instruments are used.	No	Behavior 0
		Yes	Behavior 1
16	Mandatory training on person-centred care is required for all staff.	No	Training 0
		Yes	Training 1
17	One staff member is an expert in person-centred care (and both continuously educates himself/herself on this topic and supports others with respect to its implementation).	No	Expert 0
		Yes	Expert 1
18	Dementia care mapping is conducted regularly (at least once per year) in the care unit by a person who does not work in the care unit.	No	DCM 0
		Yes	DCM 1
19	Music therapy is offered at regular intervals (at least once per week) (prior to the COVID-19 pandemic) in the residential area by a trained music therapist.	No	Music 0
		Yes	Music 1

## 2 Objective

The aim of this study was to investigate and describe differences and commonalities in different types of care units of German nursing homes regarding aspects of person-centred dementia care in terms of policies and mission statements using a mixed methods approach.

The specific study aims are (1) to investigate the differences and commonalities in the existence of policies regarding person-centred dementia care measured with the Dementia Policy Questionnaire in different types of care units in German nursing homes; (2) to explore whether policies reflect the aims and values of the mission statements regarding the different types of care units in German nursing homes; and (3) to integrate quantitative and qualitative results to compare and explain the results regarding the first objective with those regarding the second objective.

## 3 Design

**Fig. 1:**
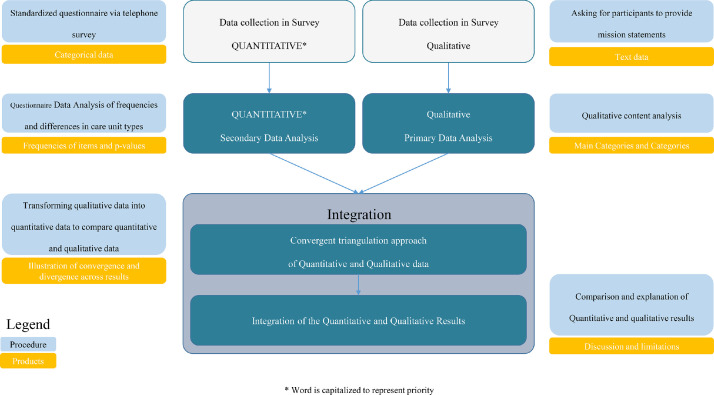
Convergent mixed methods design.

This study used a convergent mixed methods design (Creswell and Creswell, 2022; Creswell and Plano Clark, 2018). We chose this design (1) to gain a deeper understanding of policies and mission statements regarding person-centred dementia care in different types of care units in German nursing homes and (2) to explore consistency in policies about person-centred dementia care and mission statements across nursing homes with different types of care units. We analyzed the data from the BeStaDem survey, which was conducted from February 2020 to May 2021 and was performed by German Center for Neurodegenerative Diseases (DZNE), site Witten. The BeStaDem survey is a national cross-sectional study with a stratified randomized sample of 134 nursing homes in Germany that aims to develop a typology of care units (Hoffmann et al., 2021). We analyzed the data from June to September 2022. According to a convergent mixed methods design (Creswell and Creswell, 2022), quantitative cross-sectional data and qualitative documentary data on mission statements were collected in parallel in the BeStaDem survey. We analyzed these data separately. To validate the quantitative results with the qualitative results, we integrated both results by using a convergent triangulation approach (Turner et al., 2017). For integration, we transformed the qualitative data into quantitative data to compare quantitative and qualitative data and to determine whether they confirmed or disconfirmed each other (Creswell and Creswell, 2022). The quantitative data were given priority, whereas the qualitative data were used for comparison and explanation (Kuckartz, 2014). Fig. 1 illustrates the study design.

### 3.1 Setting

The BeStaDem survey included licensed nursing homes that were selected from a list (Pflegemarkt.com, 2021) containing all German nursing homes, information about the provision of a Dementia Special Care Unit and contact details.

### 3.2 Sampling

The list was stratified by federal state and the provision of a Dementia Special Care Unit and randomly sorted. To ensure that the sample distribution corresponded to all nursing homes in Germany (80 % without and 20 % with a Dementia Special Care Unit), eight nursing homes without a Dementia Special Care Unit and two with a Dementia Special Care Unit were selected per federal state. The sample size calculation was based on feasibility and the selection of an equal number of nursing homes per federal state since statistics for typology development do not require a specific sample size. Due to the lack of list reliability, nursing homes with a Dementia Special Care Unit were oversampled. The selected nursing homes were contacted by mail and phone. When a randomly selected nursing home refused to participate, the next nursing home was contacted according to the randomly sorted list (Bergmann et al., 2023).

### 3.3 Participants

In total, 1207 nursing home administrators or their representatives (nursing home managers, care unit managers of the participating nursing home) were contacted. Of these, 134 gave their informed consent (Bergmann et al., 2023).

### 3.4 Researcher description

ALH is a registered nurse and research associate who has previously conducted both quantitative and qualitative research. As part of the BeStaDem survey research team, she collected the BeStaDem survey data and analyzed the Dementia Policy Questionnaire data to explore construct validity (Hoffmann et al., 2022). MRM is also a registered nurse and research associate who is very experienced in qualitative research.

### 3.5 Data collection

#### 3.5.1 Quantitative data

In the BeStaDem survey, between June and December 2020, quantitative data were collected via computer-assisted telephone interviews with a standardized questionnaire.

#### 3.5.2 Measures

The standardized questionnaire of the BeStaDem survey asked for the provision of different contextual information (Hoffmann et al., 2021):

- At the nursing home level as well as the care unit level: structural and organizational aspects such as the number of care units with their respective names, number of beds for residents and information on whether the care unit is specialized for people living with dementia, and targeted and actual full-time positions for nurses.
- At the care unit level: Structural and organizational aspects such as (1) building design including architecture, protection by exit control, and single rooms; (2) financing including regulation and costs; (3) registered nurses including allocation, presence in the care unit and qualification; (4) number of residents including short-term care places, diagnosis of dementia, mobility, court order for accommodation, physical restraint; (5) meals including meal preparation, meal service and shared meals; and (6) changes resulting from the COVID-19 pandemic in every aspect.
- At the care unit level: The existence of internal policies about person-centred dementia care according to the Dementia Policy Questionnaire.
- Sociodemographic data of the study participants: Gender, age, working years, educational achievement, job title, and job role.

Before data collection in the BeStaDem survey, the standardized questionnaire was pretested to provide validity (Hoffmann et al., 2021). ALH, a registered nurse trained in conducting interviews, collected the data with a manual that provided information for every item and provided standardized data collection (Hoffmann et al., 2022).

#### 3.5.3 Qualitative data

As the mission statements are identified as relevant qualitative data, participants were asked to send their nursing home mission statements via email within the BeStaDem survey.

#### 3.5.4 Quantitative and qualitative data sources

The German Center for Neurodegenerative Diseases (DZNE) provided the quantitative dataset for our quantitative secondary data analysis. The German Center for Neurodegenerative Diseases (DZNE) also provided the qualitative dataset that we used as primary data for this study, as they were unprocessed after data collection.

### 3.6 Quantitative phase

#### 3.6.1 Variables and measures

For this study, to construct subgroups to measure differences in the existence of policies regarding person-centred dementia care, we used the typology of care units (Bergmann et al., 2023), which was developed from the BeStaDem survey dataset. Bergmann et al. (2023) identified the typology of care units using factor analysis of mixed data and hierarchical cluster analysis. This made it possible to define four different types of care units on the basis of the contextual characteristics collected in the survey. The variable ‘care unit type’ has four categories: (1) The Dementia Care Unit is characterized by, e.g., a self-reference as Dementia Special Care Unit, a higher percentage of people living with dementia, a special building for people living with dementia, exit controls, and a single floor. In addition to these characteristics, the (2) Dementia Special Care Unit is defined by, e.g., a specialization contractually regulated with cost bearers, more costs invested in additional staff, additional financing, and contractually regulated admission criteria. (3) The Usual Separated Care Unit is characterized by, e.g., no self-reference as a Dementia Special Care Unit, no building, especially for people living with dementia, and architectural segregation. (4) The Usual Incorporated Care Unit is characterized by, e.g., architectural integration with other care units, several floors, and no self-reference as does the Dementia Special Care Unit (Bergmann et al., 2023).

We assessed the existence of person-centred dementia care policies in German nursing homes with the Dementia Policy Questionnaire (Hoffmann et al., 2022). All 19 items were dichotomously distributed and referred to the care unit level. The Questionnaire includes three a priori subdimensions: (1) internal policies regarding recording residents’ preferences, (2) internal policies regarding participatory decision making, and (3) internal policies regarding dementia-specific interventions (Hoffmann et al., 2022) (Table 1).

#### 3.6.2 Quantitative data analysis

Regarding the first aim, ALH generated a contingency table to identify differences in the Dementia Policy Questionnaire item distributions of several care unit types. To determine whether there was a significant association between every categorical Dementia Policy Questionnaire variable and every categorical ‘care unit type’ variable, we conducted independence tests. Since in the contingency table, we identified that values in the cells were <5, we considered a suitable procedure for small sample sizes (Kim, 2017; McCrum-Gardner, 2008). We performed Fisher's exact test with the standard R Stats Package version 4.2.1, function fisher.test() (R Core Team, 2022) with two-sided hypothesis tests. H_0_ was that there is no difference in the distribution of policies on person-centred dementia care among the four care unit types. To reject H_0_, we used a cutoff α of 0.05 (Fisher, 1950). The dataset and source code in R are available from the Zenodo repository (Hoffmann et al., 2023). After a review of the BeStaDem survey dataset, we identified no missing data.

To support the results of Fisher's exact test, we additionally applied logistic regression analysis to identify associations between all four care unit types and the existence of each person-centred care policy measured with the Dementia Policy Questionnaire. In this analysis, each dichotomous person-centred dementia care policy measured with the Dementia Policy Questionnaire served as the dependent variable, and the care unit types served as the independent variable. Since the care unit type is a variable with four categories, we converted it into four dichotomous dummy variables before applying the logistic regression. We selected the USCU care unit type as the reference category for each model. We assessed the power of all the models using Nagelkerke R^2^. We considered a p value <0.05 to indicate statistical significance. Logistic regression was conducted in R (R Core Team, 2022) with the glm function.

### 3.7 Qualitative phase

#### 3.7.1 Mission statements

Of the 134 nursing homes, 60 provided their mission statements via email or they referred to their website. In addition to their mission statements, some nursing homes provided the concept of the whole nursing home or the quality management guide.

#### 3.7.2 Qualitative data analysis

For data preparation, we went to all the provided documents and identified the mission statements. We assigned mission statements to the types of care units to explore differences in addressing the policies measured with the Dementia Policy Questionnaire between nursing homes with different types of care units. We coded the mission statements using MAXQDA 22.0.1 software. To analyze whether policies reflect the aims and values of mission statements (second aim), ALH and MRM, both experienced in qualitative data analysis, deductively analyzed the mission statements by applying a qualitative content analysis method (Kuckartz and Rädiker, 2022). We used the subdimensions and items of the Dementia Policy Questionnaire as deductive categories for the qualitative content analysis.

ALH and MRM independently read the mission statements thoroughly and wrote short case summaries to become familiar with the data. During the analysis, they wrote memos. ALH generated a deductive code tree using the subdimensions of the Dementia Policy Questionnaire as the main categories and their items (Table 1) as categories. ALH generated definitions according to the item's content. To ensure quality and reliability, ALH and MRM coded concurrently based on the defined categories. To refine the deductive code tree, they first coded ten mission statements independently and discussed their results. They then specified category definitions and added text passages as examples. After refining the code tree, they coded every mission statement independently and met on a weekly basis to discuss the coding and to check them regarding accordance. They discussed different codes. If there were differences, they exchanged the reasons and, if possible, reached a consensus on the appropriate coding. If a consensus could not be reached, the research team (MR, BH, RP) was consulted to reach a consensus.

### 3.8 Integration

#### 3.8.1 Convergent triangulation approach to the quantitative and qualitative results

With respect to the third aim, we used a convergent triangulation approach (Turner et al., 2017) in which we integrated the quantitative and qualitative results. To facilitate a comparison of the quantitative results with the qualitative results, we transformed the qualitative data into quantitative categories according to Fetters et al. (2013). In tabular format, we compared the responses regarding the Dementia Policy Questionnaire items of those nursing homes whose mission statements were deductively coded according to the Dementia Policy Questionnaire subdimensions or items. This allows a clear identification of convergence and divergence across the results of the datasets (Östlund et al., 2011).

#### 3.8.2 Integration of the quantitative and qualitative results

To compare the quantitative and qualitative results (Plano Clark et al., 2010), we integrated the results into the discussion section.

#### 3.8.3 Data analysis of integration

We used the MAXQDA mixed methods tools ‘crosstable’ and ‘side-by-side display’ in MAXQDA 22.0.1 software.

### 3.9 Ethical considerations

The BeStaDem survey obtained ethical clearance from the German Society of Nursing Science in October 2018 (application number: 18-016). We met the requirements for good practice in the analysis of secondary data (AGENS, 2008, 2014).

## 4 Results

### 4.1 Characteristics of the sample

The sample included 134 nursing homes, with each nursing home providing data at the nursing home level as well as for one selected care unit. Among all the nursing homes, 29.9 % had a Dementia Care Unit, 12.7 % had a Dementia Special Care Unit, 43.3 % had a Usual Separated Care Unit, and 14.2 % had a Usual Incorporated Care Unit.

The nursing homes had a mean number of 3.3 care units. The care units had a mean size of 27.1 beds. A total of 75.6 % of all rooms in a care unit were single rooms.

A total of 60 nursing homes (44.8 %) provided either their nursing home mission statements (43) or their providers’ mission statements (17). Of those 60 nursing homes, 24 (40 %) had a Dementia Care Unit, 9 (15 %) had a Dementia Special Care Unit, 22 (36.7 %) had a Usual Separated Care Unit, and 5 (8.3 %) had a Usual Incorporated Care Unit. Of the 43 nursing homes that provided their mission statements, 15 had a Dementia Care Unit, 8 had a Dementia Special Care Unit, 16 had a Usual Separated Care Unit and 4 had a Usual Incorporated Care Unit. 17 nursing homes sent their providers’ mission statements, including 9 with a Dementia Care Unit, one with a Dementia Special Care Unit, 6 with a Usual Separated Care Unit and one with a Usual Incorporated Care Unit.

### 4.2 Quantitative findings

Regarding the investigation of the differences and commonalities in the existence of policies measured with the Dementia Policy Questionnaire among the different types of care units in German nursing homes, we identified a highly significant association (*p* < .01) between care unit types and policies (Table 2). Here, we reject H_0_. One policy concerned providing residents with all-day use of common areas (Area). Policies about the application of dementia-specific behavioral assessment instruments (Behavior), mandatory training on person-centred care (Training), and the provision of an expert on person-centred care (Expert) exist in Dementia Care Units and Dementia Special Care Units rather than in Usual Separated Care Units and Usual Incorporated Care Units. The policy concerning the provision of Dementia Care Mapping (DCM) exists in Dementia Special Care Units rather than in Dementia Care Units, Usual Separated Care Units, or Usual Incorporated Care Units.

**Table 2:** Cross-table of dementia policy questionnaire items and care unit types of German nursing homes.

Item categories (0 = nonexistent, 1 = existent)	DCU (%)	DSCU (%)	UICU (%)	USCU (%)	Margin sum (%)	*p* value
Visitor 0 (%)	40 (100.00)	17 (100.00)	19 (100.00)	56 (96.55)	132 (98.51)	0.740
Visitor 1 (%)	0 (0.00)	0 (0.00)	0 (0.00)	2 (3.45)	2 (1.49)	
Margin sum (%)	40 (100.00)	17 (100.00)	19 (100.00)	58 (100.00)	134 (100.00)	
PreferenceA 0 (%)	3 (7.50)	2 (11.76)	6 (31.58)	13 (22.41)	24 (17.91)	0.078
PreferenceA 1 (%)	37 (92.50)	15 (88.24)	13 (68.42)	45 (77.59)	110 (82.09)	
Margin sum (%)	40 (100.00)	17 (100.00)	19 (100.00)	58 (100.00)	134 (100.00)	
Sheet 0 (%)	19 (47.50)	10 (58.82)	13 (68.42)	39 (67.24)	81 (60.45)	0.217
Sheet 1 (%)	21 (52.50)	7 (41.18)	6 (31.58)	19 (32.76)	53 (39.55)	
Margin sum (%)	40 (100.00)	17 (100.00)	19 (100.00)	58 (100.00)	134 (100.00)	
Selection 0 (%)	39 (97.50)	17 (100.00)	19 (100.00)	57 (98.28)	132 (98.51)	1.000
Selection 1 (%)	1 (2.50)	0 (0.00)	0 (0.00)	1 (1.72)	2 (1.49)	
Margin sum (%)	40 (100.00)	17 (100.00)	19 (100.00)	58 (100.00)	134 (100.00)	
CConference 0 (%)	17 (42.50)	6 (35.29)	10 (52.63)	25 (43.10)	58 (43.28)	0.775
CConference 1 (%)	23 (57.50)	11 (64.71)	9 (47.37)	33 (56.90)	76 (56.72)	
Margin sum (%)	40 (100.00)	17 (100.00)	19 (100.00)	58 (100.00)	134 (100.00)	
Involvement 0 (%)	18 (45.00)	7 (41.18)	9 (47.37)	31 (53.45)	65 (48.51)	0.780
Involvement 1 (%)	22 (55.00)	10 (58.82)	10 (52.63)	27 (46.55)	69 (51.49)	
Margin sum (%)	40 (100.00)	17 (100.00)	19 (100.00)	58 (100.00)	134 (100.00)	
**Area 0 (%)**	13 (32.50)	2 (11.76)	1 (5.26)	23 (39.66)	39 (29.10)	<0.01 ***
**Area 1 (%)**	27 (67.50)	15 (88.24)	18 (94.74)	35 (60.34)	95 (70.90)	
Margin sum (%)	40 (100.00)	17 (100.00)	19 (100.00)	58 (100.00)	134 (100.00)	
PreferenceB 0 (%)	6 (15.00)	3 (17.65)	5 (26.32)	16 (27.59)	30 (22.39)	0.475
PreferenceB 1 (%)	34 (85.00)	14 (82.35)	14 (73.68)	42 (72.41)	104 (77.61)	
Margin sum (%)	40 (100.00)	17 (100.00)	19 (100.00)	58 (100.00)	134 (100.00)	
PreferenceC 0 (%)	33 (82.50)	16 (94.12)	16 (84.21)	47 (81.03)	112 (83.58)	0.716
PreferenceC 1 (%)	7 (17.50)	1 (5.88)	3 (15.79)	11 (18.97)	22 (16.42)	
Margin sum (%)	40 (100.00)	17 (100.00)	19 (100.00)	58 (100.00)	134 (100.00)	
CarePlan 0 (%)	6 (15.00)	1 (5.88)	0 (0.00)	4 (6.90)	11 (8.21)	0.272
CarePlan 1 (%)	34 (85.00)	16 (94.12)	19 (100.00)	54 (93.10)	123 (91.79)	
Margin sum (%)	40 (100.00)	17 (100.00)	19 (100.00)	58 (100.00)	134 (100.00)	
AltRestrict 0 (%)	10 (25.00)	3 (17.65)	6 (31.58)	15 (25.86)	34 (25.37)	0.838
AltRestrict 1 (%)	30 (75.00)	14 (82.35)	13 (68.42)	43 (74.14)	100 (74.63)	
Margin sum (%)	40 (100.00)	17 (100.00)	19 (100.00)	58 (100.00)	134 (100.00)	
Rejection 0 (%)	16 (40.00)	7 (41.18)	9 (47.37)	21 (36.21)	53 (39.55)	0.856
Rejection 1 (%)	24 (60.00)	10 (58.82)	10 (52.63)	37 (63.79)	81 (60.45)	
Margin sum (%)	40 (100.00)	17 (100.00)	19 (100.00)	58 (100.00)	134 (100.00)	
Drugs 0 (%)	25 (62.50)	12 (70.59)	12 (63.16)	40 (68.97)	89 (66.42)	0.896
Drugs 1 (%)	15 (37.50)	5 (29.41)	7 (36.84)	18 (31.03)	45 (33.58)	
Margin sum (%)	40 (100.00)	17 (100.00)	19 (100.00)	58 (100.00)	134 (100.00)	
Pain 0 (%)	3 (7.50)	1 (5.88)	6 (31.58)	10 (17.24)	20 (14.93)	0.075
Pain 1 (%)	37 (92.50)	16 (94.12)	13 (68.42)	48 (82.76)	114 (85.07)	
Margin sum (%)	40 (100.00)	17 (100.00)	19 (100.00)	58 (100.00)	134 (100.00)	
**Behavior 0 (%)**	26 (65.00)	6 (35.29)	16 (84.21)	50 (86.21)	98 (73.13)	<0.01 ***
**Behavior 1 (%)**	14 (35.00)	11 (64.71)	3 (15.79)	8 (13.79)	36 (26.87)	
Margin sum (%)	40 (100.00)	17 (100.00)	19 (100.00)	58 (100.00)	134 (100.00)	
**Training 0 (%)**	18 (45.00)	7 (41.18)	18 (94.74)	41 (70.69)	84 (62.69)	<0.01 ***
**Training 1 (%)**	22 (55.00)	10 (58.82)	1 (5.26)	17 (29.31)	50 (37.31)	
Margin sum (%)	40 (100.00)	17 (100.00)	19 (100.00)	58 (100.00)	134 (100.00)	
**Expert 0 (%)**	27 (67.50)	8 (47.06)	19 (100.00)	54 (93.10)	108 (80.60)	<0.01 ***
**Expert 1 (%)**	13 (32.50)	9 (52.94)	0 (0.00)	4 (6.90)	26 (19.40)	
Margin sum (%)	40 (100.00)	17 (100.00)	19 (100.00)	58 (100.00)	134 (100.00)	
**DCM 0 (%)**	36 (90.00)	9 (52.94)	19 (100.00)	52 (89.66)	116 (86.57)	<0.01 ***
**DCM 1 (%)**	4 (10.00)	8 (47.06)	0 (0.00)	6 (10.34)	18 (13.43)	
Margin sum (%)	40 (100.00)	17 (100.00)	19 (100.00)	58 (100.00)	134 (100.00)	
Music 0 (%)	34 (85.00)	14 (82.35)	17 (89.47)	56 (96.55)	121 (90.30)	0.092
Music 1 (%)	6 (15.00)	3 (17.65)	2 (10.53)	2 (3.45)	13 (9.70)	
Margin sum (%)	40 (100.00)	17 (100.00)	19 (100.00)	58 (100.00)	134 (100.00)	

DCU Dementia Care Unit.

DSCU Dementia Special Care Unit.

UICU Usual Incorporated Care Unit.

USCU Usual Separated Care Unit.

The numbers represent absolute frequencies.

The numbers in brackets () represent the relative frequencies as percentages of the respective care unit type possessing the corresponding category. Example: Two out of 17 care units in the DSCU type have the category PreferenceA 0, that is, 11.8 percent.

p value: Independent Fisher's test comparing care unit types of German nursing homes.

**p < .05, ***p < .01.

The logistic regression analysis supported the results of Fisher's exact test. Table 3 shows the significant results for the associations between care unit types as independent variables and person-centred care policies as dependent variables. Compared with the Usual Separated Care Unit, Usual Incorporated Care Units (odds ratio 11.83) and Dementia Special Care Units (odds ratio 4.93) were more likely to provide a policy about all-day use of common areas (Area).

**Table 3:** Logistic regression - associations between care unit types and the existence of person-centred care policies measured with the dementia policy questionnaire.

Dependent variable	Predictor	Co-efficient β	Standard error	Wald test	*P* value	Odds ratio OR	Confidence interval CI (2.5 %)	Confidence interval CI (97.5 %)
**Area**	Intercept	0.4199	0.2684	1.5642	0.1178	1.5217	0.9051	2.6082
ChiSquare Omnibus-Test: 0.0044	**UICU**	2.4705	1.0619	2.3266	0.0200**	11.8286	2.2013	220.0612
Nagelkerke R^2^: 0.1332	**DCU**	0.3110	0.4313	0.7212	0.4708	1.3648	0.5906	3.2319
	**DSCU**	1.5950	0.7992	1.9958	0.0460**	4.9286	1.2345	33.1554
**Behavior**	Intercept	-1.8326	0.3808	-4.8126	0.0000***	0.1600	0.0701	0.3185
ChiSquare Omnibus-Test: 0.0003	**UICU**	0.1586	0.7354	0.2157	0.8292	1.1719	0.2356	4.6194
Nagelkerke R^2^: 0.192	**DCU**	1.2135	0.5049	2.4037	0.0162**	3.3654	1.2761	9.4209
	**DSCU**	2.4387	0.6345	3.8436	0.0001***	11.4583	3.4471	42.5729
**Training**	Intercept	-0.8804	0.2885	-3.0518	0.0023***	0.4146	0.2295	0.7166
ChiSquare Omnibus-Test: 0.0001	**UICU**	-2.0100	1.0671	-1.8836	0.0596	0.1340	0.0072	0.7327
Nagelkerke R^2^: 0.1974	**DCU**	1.0810	0.4292	2.5186	0.0118**	2.9477	1.2839	6.9535
	**DSCU**	1.2370	0.5710	2.1663	0.0303**	3.4454	1.1404	10.9761
**Expert**	Intercept	-2.6027	0.5182	-5.0227	0.0000***	0.0741	0.0224	0.1806
ChiSquare Omnibus-Test: 0	**UICU**	-15.9634	1496.3960	-0.0107	0.9915	0.0000	0.0000	—
Nagelkerke R^2^: 0.3088	**DCU**	1.8718	0.6184	3.0266	0.0025**	6.5000	2.0795	24.8402
	**DSCU**	2.7205	0.7104	3.8296	0.0001***	15.1875	4.0127	68.1694
**DCM**	Intercept	-2.1595	0.4312	-5.0086	0.0000***	0.1154	0.0443	0.2478
ChiSquare Omnibus-Test: 0.0005	**UICU**	-16.4066	1496.3960	-0.0110	0.9913	0.0000	—	—
Nagelkerke R^2^: 0.226	**DCU**	-0.0377	0.6809	-0.0554	0.9558	0.9630	0.2322	3.6135
	**DSCU**	2.0417	0.6496	3.1429	0.0017***	7.7037	2.2003	29.0163

DCU Dementia Care Unit.

DSCU Dementia Special Care Unit.

UICU Usual Incorporated Care Unit.

USCU Usual Separated Care Unit.

**p < .05, ***p < .01.

The Dementia Care Unit (odds ratio 3.37) and the Dementia Special Care Unit (odds ratio 11.46) are more likely to provide policies about the application of dementia-specific behavioral assessment instruments (Behavior) compared to the Usual Separated Care Unit. We identified a similar observation for two other policies measured with the Dementia Policy Questionnaire: (1) The existence of policies about mandatory training on person-centred care (Training), where Dementia Care Units (odds ratio 2.95) and Dementia Special Care Units (odds ratio 3.45) are more likely to provide these policies than the Usual Separated Care Unit type. (2) The existence of policies concerning the provision of an expert on person-centred care (Expert), where the Dementia Care Units (odds ratio 6.50) and Dementia Special Care Units (odds ratio 15.19) are more likely to provide these policies than the Usual Separated Care Unit type. Compared to the Usual Separated Care Unit, the Dementia Special Care Unit (odds ratio 7.70) is more likely to provide Dementia Care Mapping policies.

For the power of all the models measured with Nagelkerke R^2^, Table 3 shows that the model with the dependent variable 1) Area (0.13) has poorer model quality, whereas the models with the dependent variables 2) Behavior (0.19), 3) Training (0.20), 4) Expert (0.31), and 5) Dementia Care Mapping (0.23) have acceptable effects.

### 4.3 Qualitative findings

To explore whether the policies measured with the Dementia Policy Questionnaire were addressed in mission statements, we examined 60 mission statements. We identified segments of the Dementia Policy Questionnaire items exclusively in the mission statements of nursing homes with Dementia Care Units and Dementia Special Care Units. We coded four deductive categories in four provider mission statements and four nursing home mission statements:

#### 4.3.1 Visitor regulations

We coded ‘visitor regulations’ in one provider mission statement from a nursing home with a Dementia Care Unit. Visitor regulation was described by specifying that there were no specific times or number of visits: *“Visits are possible at any time”* (Nursing home 112 with Dementia Care Unit).

#### 4.3.2 Care plan

We coded ‘care plan’ in five mission statements of nursing homes providing a Dementia Care Unit, two with a provider and three with a nursing home mission statement as well as in two nursing home mission statements of a nursing home providing a Dementia Special Care Unit. The mission statements described the involvement of residents and their significant others in care planning. While one mission statement of a nursing home with a Dementia Care Unit stated that care planning was carried out together with relatives “if possible” (nursing home 21 with Dementia Care Unit), one mission statement of the nursing home with a Dementia Special Care Unit described their involvement as linked to the resident's preferences. One mission statement described aspects of care planning as follows:

“The focal points in the care are planned by the employees with the residents […] and/or their relatives/reference persons, by accounting for the social environment, personal needs, biography, individual risks and the therapeutic-promoting approaches.” (Nursing home 102 with Dementia Special Care Unit).

#### 4.3.3 Alternative procedures to restricting measures

We coded this category in one NH mission statement of a nursing home providing a Dementia Special Care Unit that addressed protection from measures that restrict freedom:

“We protect the residents […] from violence, measures that restrict freedom and neglect.” (Nursing home 102 with Dementia Special Care Unit).

#### 4.3.4 Dementia-specific interventions

We coded this main category in two provider mission statements of nursing homes with a Dementia Care Unit. They described the provision of specialized care exclusively for people living with dementia without describing which kind of interventions. *“We offer residents suffering from dementia specialized care and support that relieves them and is appropriate for their special situation.”* (Nursing home 113 with Dementia Special Care Unit).

#### 4.3.5 Expert in person-centred care

One nursing home mission statement of a nursing home with a Dementia Care Unit that addressed the person-centred care model by Tom Kitwood described the existence of special qualified staff:

*“We rely on competent staff with specialized training. To this end, we attach particular importance to further training in the specialist field of gerontological psychiatry.”* (Nursing home 126 with Dementia Care Unit).

### 4.4 Convergent triangulation approach to the quantitative and qualitative results

Regarding the integration of the quantitative and qualitative results, we transformed the qualitative data into quantitative categories to compare the quantitative and qualitative results regarding divergence and convergence. Table 4 shows a comparison of the responses regarding the Dementia Policy Questionnaire items of those nursing homes whose mission statements were deductively coded according to the Questionnaire subdimensions or items.

**Table 4:** Triangulation of the quantitative and qualitative results.

	Dementia-specific interventions		Expert in PCC	Alternative procedures to restricting measures	Visitor regulations	Care plan						
	NH 112 with DCU	NH 53 with DCU	NH 126 with DCU	NH 102 with DSCU	NH 112 with DCU	NH 3 with DSCU	NH 102 with DSCU	NH 21 with DCU	NH 11 with DCU	NH with 126 DCU	NH 9 with DCU	NH 102 with DCU
	PRO	PRO	NH	NH	PRO	NH	NH	PRO	NH	NH	PRO	NH
Visitor	–	–	–	–	**- -**	–	–	–	–	–	–	–
PreferenceA	+	+	–	+	+	+	+	+	+	–	+	+
Sheet	+	–	+	+	+	+	+	–	+	+	+	+
Selection	–	–	–	–	–	–	–	–	–	–	+	–
CConference	+	+	+	+	+	+	+	–	+	+	+	+
Involvement	+	–	+	+	+	+	+	–	+	+	+	+
Area	–	+	+	+	–	+	+	+	–	+	+	+
PreferenceB	+	+	+	+	+	+	+	+	+	+	+	+
PreferenceC	–	–	+	–	–	+	+	–	–	+	+	+
CarePlan	+	+	+	+	+	**+++**	**+++**	**+++**	**+++**	**+++**	**+++**	**+++**
AltRestrict	+	+	+	**+++**	+	+	+	+	+	+	+	+
Rejection	+	+	+	+	+	+	+	+	+	+	+	+
Drugs	**+++**	**+++**	–	–	+	–	–	–	–	–	+	–
Pain	**+++**	**+++**	+	+	+	+	+	+	+	+	+	+
Behavior	**+++**	**+++**	–	+	+	+	+	+	–	–	–	+
Training	**+++**	**–**	+	–	+	+	–	+	+	+	–	+
Expert	**+++**	**+++**	**—**	+	+	+	+	–	+	–	–	+
DCM	**+++**	**—**	–	+	+	+	+	–	–	–	–	–
Music	**—**	**+++**	–	+	–	–	+	–	–	–	–	–
Convergence/Divergence	Divergence		Divergence	Convergence	Convergence	Convergence						

PCC= Person-centred care.

NH= Nursing Home.

DCU= Dementia Care Unit.

DSCU= Dementia Special Care Unit.

PRO= Provider mission statement.

NH= Nursing Home Mission Statement.

–= nonexistent.

+= existent.

+++= existent and coded in mission statements.

—= nonexistent but coded in mission statements.

#### 4.4.1 Triangulation of divergent results

According to Table 4, divergence was found in mission statements addressing dementia-specific interventions and person-centred care experts. The main deductive category ‘dementia-specific intervention’ was coded in two mission statements of nursing homes with a Dementia Care Unit. As a Dementia Policy Questionnaire subdimension, there is no item that assesses policies about dementia-specific interventions in general. Although these mission statements provided most of the policy items assigned to this subdimension, nursing home 112 reported not having a music therapy policy, and nursing home 53 said they do not have a Dementia Care Mapping (DCM) policy. One mission statement of a nursing home with a Dementia Care Unit pointed out that they had employed a person-centred care expert while the equivalent item was negatively responded to in the survey.

#### 4.4.2 Triangulation of convergent results

Convergence was found in nursing homes that included content in their policies linked to alternative procedures to restricting measures, visitor regulation, and care planning and that addressed these aspects in their mission statements. The deductive code ‘alternative procedures to measures that restrict freedom’ was addressed in a mission statement of one nursing home with a Dementia Special Care Unit, which corresponded to their answer in the survey. A nursing home with a Dementia Care Unit that reported that visitors are welcome at any time in their mission statement did not have a visitor policy. Two nursing homes with a Dementia Special Care Unit and five nursing homes with a Dementia Care Unit addressed joint care planning in their mission statements, and all answered the equivalent in the survey (Table 4).

## 5 Discussion

This convergent mixed methods study aimed to (1) identify the differences and commonalities in the existence of policies about person-centred dementia care across four care unit types in German nursing homes, (2) explore whether the policies measured with the Dementia Policy Questionnaire are addressed in mission statements, and (3) integrate the quantitative and qualitative results. The quantitative results revealed that Dementia Care Units and Dementia Special Care Units provide more policies on person-centred dementia care (Table 2). The qualitative results revealed that the person-centred dementia care policies of nursing homes are overall not consistent with the goals addressed in their mission statements. Only 8 out of 60 mission statements exclusively of nursing homes with a Dementia Care Unit or Dementia Special Care Unit address single aspects that are measured with the Dementia Policy Questionnaire. The convergent triangulation approach revealed that, except for the subdimension of dementia-specific interventions and the person-centred care expert item, the qualitative results converged with the response pattern of the nursing home survey.

The quantitative results show a highly significant association (*p*value < 0.01) between the items assessing policies about behavior assessment, person-centred care training, person-centred care expert, Dementia Care Mapping and care unit types, which mostly exist in the Dementia Care Unit and Dementia Special Care Unit types. This finding supports the results of Bergmann et al. (2023). The application of dementia-specific behavior assessments is required by separate supply arrangements for Dementia Special Care Units (Stadt Hamburg, 2016). Policies about Dementia Care Mapping (DCM) mostly exist in Dementia Special Care Units, and person-centred care staff training mostly exists in Dementia Care Units and Dementia Special Care Units. These interventions have been recommended as nonpharmacological interventions for managing Behavioral and Psychological Symptoms of Dementia (BPSD) (Backman et al., 2021; Chenoweth et al., 2009; Jutkowitz et al., 2016; Livingston et al., 2018). Person-centred care training refines one's own perspective and awareness of person-centred care as well as the handling of responsive behavior and antipsychotic use (Fossey et al., 2006; Richter et al., 2022). Our qualitative data confirm that dementia-specific interventions are addressed in two mission statements. For the person-centred care expert category, one nursing home with a Dementia Care Unit claimed not to provide a person-centred care expert in the survey but described the specialization of staff in their mission statement. We identified one mission statement of a nursing home with a Usual Separated Care Unit that mentioned the provision of gerontological-psychiatric nurse specialists. In Germany, gerontological-psychiatric nurse training also includes knowledge transfer in person-centred care. It is assumed that even Usual Separated Care Units and Usual Incorporated Care Units have a high demand for gerontological-psychiatric skills (Wingenfeld, 2012). Nevertheless, it is unclear whether these specialists fulfill the function of person-centred care experts.

The quantitative results show that in all care unit types, except in six Dementia Care Units, policies about joint care planning exist. This contradicts the qualitative results, which show that care planning is addressed exclusively in the mission statements of nursing homes with Dementia Care Units and Dementia Special Care Units. Joint care planning with residents and relatives to prioritize knowledge about, e.g., unique routines, preferences, and the maintenance of independence, is considered individualized care (Molony et al., 2018). McCreedy et al. (2018) also identified in their sample that for 56 % of residents with severe cognitive impairment, neither a relative nor themselves participated in care planning. We assume that in nursing homes with Dementia Care Units and Dementia Special Care Units, joint care planning might not always be implemented due to challenging communication with the resident, where the justification of joint care planning is deemed less relevant and is therefore not recorded in the form of policies.

In our quantitative analysis, the item used to assess the existence of policies about alternative procedures to restraints was not significant. Table 2 shows that Dementia Special Care Units most frequently provide these policies. As shown in the qualitative analysis, one mission statement of a nursing home with a Dementia Special Care Unit addresses this procedure. Bergmann et al. (2023) showed that 52.9 % of all Dementia Special Care Units in the sample were protected by exit controls, whereas only 16.4 % of all care units in the sample provided exit controls. Considering that these controls are a form of freedom restriction (Köpke et al., 2015) contrasts our results. Our results are in line with the conclusions of Sloane et al. (1991), who explain the low rate of physical restraint use with the provision of regulations and practices in Dementia Special Care Units.

The results of this study have implications for changes needed within the organizational policy development processes as well as practice adaptations: According to our results, we worry that nursing homes (despite the care unit type), might not adjust the development of internal policies with their vision, purpose and strategic direction addressed in their mission statements. This assumption corresponds with the identified scepticism and general discussion regarding the effectiveness of mission statements in organizations. In general, mission statements (1) might be developed in a top-down manner, (2) they might be very clearly written, and (3) they might not be implemented in practice (Alegre et al., 2018; Braun et al., 2012; Mullane, 2002). Other authors recommend the need to align internal policies while focusing on the objectives of the mission statement (Braun et al., 2012; Hoyle, 2017). This requires, for example, that mission statements are clearly formulated, that several stakeholders are included in the development processes, and that mission statements are communicated with employees within the organization. Only then will a mission statement come to life in care practices (Alegre et al., 2018). It can be assumed that some of the nursing homes need to update their mission statements, specifically those mission statements that do not address any aspect measured by the Dementia Policy Questionnaire, but where the participants in the survey indicated that PCDC policies exists. It is recommended that organizations develop and update their mission statements over time and or according to changes concerning the organization (Alegre et al., 2018; Koch et al., 2014).

The results of our study also suggest several aspects for future research questions regarding person-centred dementia care in nursing homes. Future research needs to address the following topics: (1) how do policies and mission statements relate to aspects of person-centred dementia care, (2) how are mission statements and internal policies developed within a nursing home, (3) how do mission statements and policies affect the actual implementation of person-centred dementia care, (4) which other aspects of person-centred dementia care are not measured with the questionnaire, but are mentioned in mission statements, and (5) what is highly relevant for nursing homes when developing their mission statements and internal policies*.*

## 6 Limitations

Regarding limitations, it was not possible to conduct a sequential mixed methods design or to consider more integration strategies for quantitative and qualitative data. With convergent triangulation, we increased the trustworthiness of our initial hypothesis. Nonetheless, there is an unequal sample size problem in the quantitative and qualitative data since in the BeStaDem survey, not all 134 nursing homes provided their mission statements. Although a contrasting analysis of different types of care units – based on the qualitative data – was possible, we consider the comparison of policies (at the care unit level) with mission statements (at the nursing home or provider level) a challenging task.

## 7 Conclusions

Our study provides an overview of German nursing homes regarding the existence of internal policies about person-centred dementia care assessed with the German Dementia Policy Questionnaire and mission statements across different types of care units. The results show that nursing homes with Dementia Care Units and Dementia Special Care Units have more policies, especially regarding dementia-specific interventions, and mission statements that address person-centred dementia care than do nursing homes with Usual Incorporated Care Units and Usual Separated Care Units. We assume that the Dementia Policy Questionnaire serves as an instrument to show the number of existing person-centred dementia care policies in different types of care units. Nonetheless, we did not identify a connection between these policies and mission statements. From an organizational perspective, policies should be consistent with the purpose of an organization and should therefore be deduced from the aims and principles described in mission statements (Hoyle, 2017). We conclude that nursing homes should generate mission statements to provide a clear foundation for person-centred dementia care.

## Use of data in previous publications

1. Hoffmann AL, Bergmann JM, Mueller-Widmer R, Palm R. Dementia specific care structures in nursing homes—Study protocol of a telephone-based survey study in a nationwide random sample. Journal of Advanced Nursing. 2021;n/a(n/a) :https://org/10.1111/jan.14873
2. Hoffmann AL, Bergmann JM, Fahsold A, et al. Measuring person‑centred care in german nursing homes – exploring the construct validity of the Dementia Policy Questionnaire: a cross‑sectional study of a secondary data set. BMC Geriatrics. 2022;22(914):1-12. :https://org/10.1186/s12877-
3. Bergmann JM, Hoffmann AL, Müller-Widmer R, Palm R. Typology of Dementia-Specific Care Units: A Nationwide Survey Study in Germany. Innovation in Aging. 2023;10.1093/geroni/igad062

## Funding sources

The 10.13039/501100001736German Research Foundation (DFG, project number: 430919791) funded the data collection of the secondary dataset. The German Center for Neurodegenerative Diseases (DZNE) (grant number N/A) funded the data analysis of this study as well as manuscript draft. The funders were not involved in the research process or manuscript writing.

## Data availability

Access to the qualitative data is possible upon request to the German Center for Neurodegenerative Diseases (DZNE), site Witten, Germany. Please contact the data management of the German Center for Neurodegenerative Diseases (DZNE), site Witten (data-management-witten(at)dzne.de). The quantitative dataset generated and/or analyzed for the current study and the R code are available in the Zenodo repository, DOI 10.5281/zenodo.8136635.

## CRediT authorship contribution statement

**Anna Louisa Hoffmann-Hoffrichter:** Writing – review & editing, Writing – original draft, Visualization, Validation, Supervision, Software, Project administration, Methodology, Investigation, Formal analysis, Data curation, Conceptualization. **Mike Rommerskirch-Manietta:** Writing – review & editing, Validation, Methodology. **Johannes Michael Bergmann:** Writing – review & editing, Validation, Methodology. **Martina Roes:** Writing – review & editing, Validation. **Bernhard Holle:** Writing – review & editing. **Rebecca Palm:** Writing – review & editing.

## Declaration of competing interest

The authors declare that they have no known competing financial interests or personal relationships that could have appeared to influence the work reported in this paper.
